# An ensemble classification method based on machine learning models for malicious Uniform Resource Locators (URL)

**DOI:** 10.1371/journal.pone.0302196

**Published:** 2024-05-31

**Authors:** Suresh Sankaranarayanan, Arvinthan Thevar Sivachandran, Anis Salwa Mohd Khairuddin, Khairunnisa Hasikin, Abdul Rahman Wahab Sait

**Affiliations:** 1 Department of Computer Science, King Faisal University, Al Ahsa, Kingdom of Saudi Arabia; 2 Department of Electrical Engineering, Faculty of Engineering, Universiti Malaya, Kuala Lumpur, Malaysia; 3 Department of Biomedical Engineering, Faculty of Engineering, Universiti Malaya, Kuala Lumpur, Malaysia; 4 Centre of Intelligent Systems for Emerging Technology (CISET), Faculty of Engineering, Universiti Malaya, Kuala Lumpur, Malaysia; 5 Department of Documents and Archive, Centre of Documents and Administrative Communication, King Faisal University, Al Ahsa, Kingdom of Saudi Arabia; Universiti Malaysia Sabah, MALAYSIA

## Abstract

Web applications are important for various online businesses and operations because of their platform stability and low operation cost. The increasing usage of Internet-of-Things (IoT) devices within a network has contributed to the rise of network intrusion issues due to malicious Uniform Resource Locators (URLs). Generally, malicious URLs are initiated to promote scams, attacks, and frauds which can lead to high-risk intrusion. Several methods have been developed to detect malicious URLs in previous works. There has been a good amount of work done to detect malicious URLs using various methods such as random forest, regression, LightGBM, and more as reported in the literature. However, most of the previous works focused on the binary classification of malicious URLs and are tested on limited URL datasets. Nevertheless, the detection of malicious URLs remains a challenging task that remains open to research. Hence, this work proposed a stacking-based ensemble classifier to perform multi-class classification of malicious URLs on larger URL datasets to justify the robustness of the proposed method. This study focuses on obtaining lexical features directly from the URL to identify malicious websites. Then, the proposed stacking-based ensemble classifier is developed by integrating Random Forest, XGBoost, LightGBM, and CatBoost. In addition, hyperparameter tuning was performed using the Randomized Search method to optimize the proposed classifier. The proposed stacking-based ensemble classifier aims to take advantage of the performance of each machine learning model and aggregate the output to improve prediction accuracy. The classification accuracies of the machine learning model when applied individually are 93.6%, 95.2%, 95.7% and 94.8% for random forest, XGBoost, LightGBM, and CatBoost respectively. The proposed stacking-based ensemble classifier has shown significant results in classifying four classes of malicious URLs (phishing, malware, defacement, and benign) with an average accuracy of 96.8% when benchmarked with previous works.

## 1 Introduction

The application of Internet of Things (IoT) devices is increasing and such proliferation of IoT devices has contributed to the increment of security vulnerabilities through malicious uniform resource locators (URLs). This is because devices that shared IoT networks adopted similar URLs. URL is established by the protocol applied to detect and open a specific resource in the shared IoT network. Hence, malicious URL attacks can lead to high risks of invasion, such as losses of personal records, financial damages, and data thefts. The web is essential for information exchange between people and organizations. Insecure web servers are very easy for an attacker to take over. The malicious uniform resource locator (URL) that directs users to attack-conducting web pages is dispersed across a variety of social networks and widely used web applications, social networks and game websites. Furthermore, malicious websites are the main attack vendors that cybercriminals employ to disseminate malware especially when a person visits a malicious website. Therefore, to ensure cybersecurity, there is an important need to detect malicious websites [1, 2].

The types of malicious URLs include phishing, malware, and defacement. Phishing is one of the most popular assaults where the primary goal is to fool the user into providing their information, typically login information. There are various methods for phishing, but one of them is to purchase a domain similar to the site that they want to duplicate, for example, facebok.com instead of facebook.com. Therefore, the URLs that lead to these phishing sites are known as phishing URLs. The second type of malicious URL is the malware URL. Malware includes computer viruses, worms, trojan horses, and spyware that can lead to malicious events including information theft, data encryption, deletion of sensitive data, hijacking of core computing functionalities, and illegal monitoring of user activities on computers. The malware could then corrupt files and programs in their computer. Thirdly, a defacement URL leads to an online defacement attack that occurs when a fraudster breaks into a website and changes its content with different messages that may include vulgar language or other abusive messages. Unexpected modifications to the files that are saved on the website could signify a defacement assault and a security breach [3–5].

Previous works have developed various solutions to identify malicious URLs which can be classified into three classes: blacklists, content-based classification, and URL-based classification. ’’Blacklists’’ indicates the techniques that utilize records of possible malicious URLs to filter the new URLs. Albeit blacklists can be updated regularly, this technique may ignore new malicious sites that are constantly generated. Secondly, content-based classification attempts to detect malicious sites by examining the pages’ contents or layouts. Unfortunately, fraudsters will continuously attempt new methods and use more advanced countermeasures. Hence, the content-based detecting method will usually fail. Meanwhile, as a basic setup, URL plays a vital role in an online business. The ’’background’’ data of a URL is more dependable to recognize malicious sites compared to the page’s content because such information is challenging to manipulate by fraudsters.

Currently, the adoption of machine learning models on malicious URLs is one of the main ways to classify them and overcome the short-coming of blacklisting and content-based approach. Machine learning techniques have become increasingly popular among researchers in recent years for use in a variety of cybersecurity applications, including intrusion detection, harmful URL classification, anomaly detection, and cybercrime classification. Existing works that applied machine learning methods mostly focused on binary classification of malicious URLs instead of multi-class classification. Multi-class classification is more challenging to solve due to the complex features that differentiate between types of malicious URLs. The classification challenge of malicious URL detection entails intricate capabilities such as data gathering, feature extraction, pre-processing, and classification [3–7].

In this research, a feature engineering approach is adopted to extract vital information from the URLs. Then, a stacked ensemble classifier is developed to classify malicious URLs into four classes namely phishing, benign, malware, and defacement. To optimize the model, hyperparameter tuning was performed using the Randomized Search method. The contributions of this research are as follows:

1. Development and validation of a stacked ensemble classifier by stacking four machine learning algorithms- Random Forest, XGBoost, LightGBM, and CatBoost
2. Validation of machine learning algorithms- Random Forest, XGboost, LightGBM, CatBoost
3. Comparative analysis of the stacked ensemble classifier against other machine learning algorithms.

This work acts as the starting platform for researchers to develop cyber-security solutions for IoT devices. Validations were performed to justify the contribution of the proposed method and compared against the state-of-the-art models. The paper is organised as follows. Section 2 provides detailed literature review pertaining to the usage of machine learning towards malicious URL detection. Section 3 discusses the implementation methodology of this work which includes description of dataset, feature engineering and data pre-processing. Section 4 presents the proposed methodology in this work which is the stacking-based ensemble classifier. Section 5 discusses the results and analysis of the proposed work against previous works. Section 6 gives the concluding remarks and future works.

## 2 Related works

The research work in [3] applied LightGBM and features of the domain name to recognize phishing websites and preserve the website’s security. After the filtering process, sixteen features of the domain name were extracted for the training process. The grid-search method was applied in the training process to optimize the parameters of the LightGBM model. The work achieved a classification accuracy of 93.88%. The work was tested on a limited dataset of 24000 URLs and only focused on classifying between phishing and non-phishing classes.

Researchers in [4] developed Artificial Fish Swarm Algorithm (AFSA) with deep learning model to identify malicious URLs. The proposed model executes data pre-processing while the developed vector model is fed to the Gated recurrent units (GRU) classification model. Albeit the high classification accuracy of 99.6%, this work focused on binary classification only and was tested on a limited dataset of 10,000 URLs only.

The work in [5] developed two layers of detection. Initially, the URLs are identified as either benign or malware using a binary classifier. Then, the URL is classified based on its features into five classes. The model achieved a classification accuracy of 97.9% and was tested on a limited dataset of 57000 URLs only.

Research work in [6] proposed a solution to identify fraudulent URLs in social networks like Twitter. The work categorized the URLs into four groups which are lexical elements, web content, host-based features, and popularity features. In the study, three machine learning algorithms were applied which are Logistic Regression, SVM, and Random Forest, and achieved an accuracy of 90.28%, 93.43%, and 95.51% respectively. The work was tested on a limited dataset of 11054 URLs only and only focused on classifying between phishing and non-phishing classes.

The study was conducted in [7] to detect malicious URLs, a binary classification method with an accuracy of 89%. This work was tested on a limited dataset of 5,000 URLs and only focused on classifying between malicious and benign classes.

The work in [8] presented a feature selection-based deep learning model. The work compared several classifier algorithms, including KNN, Random Forest, deep learning, and Random Forest (RF). The results showed that Random Forest is the most suitable model for deployment as compared to other methods with an accuracy of 96.26%. The work was tested on a limited dataset of 18982 URLs only. Hence, the proposed method may be less robust for the larger dataset. Besides that, the work applied a single classifier which is a random forest which can be less effective when tested on larger and more complex URL datasets.

The work in [9] proposed a feature engineering approach for detecting malicious URLs detection. The results showed that the proposed feature engineering method is effective and could improve the performances of certain classifiers in recognizing malicious URLs. The work implemented the KNN classifier and achieved an accuracy of 93.05% when tested on 331622 URLs. However, the work is limited to a simple classification problem which is to classify between benign and malicious.

The work in [10] proposed a method to detect phishing by applying machine learning algorithms. The work focused on the behaviors and qualities of the URL. The work applied the LightGBM classifier to perform binary classification. The work achieved the highest accuracy of 86%. However, the work was tested on a limited dataset of 38300 URLs for a simple classification problem which is to classify between phishing and benign classes. Table 1 summarizes the previous works on malicious URL detection in terms of method applied, dataset, advantage of the work and limitation of the work.

**Table 1:** Summary of previous works on malicious URL detection.

Work	Method	Dataset	Advantage	Limitation of the study
[3]	Grid-search optimization method and LightGBM classifier with accuracy of 93.88%.	Total URLs of 24000 from PhishTank and Alexa websites.	The proposed model outperformed GBDT, AdaBoost, XGBoost, and SVM models.	Classification for 2 classes only and used limited dataset.
[4]	A novel deep learning model named AFSADL-MURLC with accuracy of 99.6%.	Total URLs of 10000 from Kaggle repository	The proposed model outperformed RF, NB, MLP and LSTM models.	Classification for 2 classes only and tested on limited dataset.
[5]	Bagging Decision Tree Ensemble method with accuracy of 97.92%.	Total URLs of 57000 from ISCX-URL2016	Classification for 5 classes. The proposed model outperformed RF, NB, MLP and KNN models.	Tested on limited dataset.
[6]	Logistic Regression with accuracy of 90.28%, SVM with accuracy of 93.43% and Random Forest with accuracy of 95.51%	Total URLs of 11054 from PhishTank database	The classification model can detect tweets containing a phishing URL in real-time.	Classification for 2 classes only and tested on limited dataset.
[7]	KNN using random subspace technique with accuracy of 89.24%.	Total URLs of 5000 from private dataset	Comparative evaluation of several machine learning algorithms and ensemble techniques for classification.	Classification for 2 classes only and small dataset was tested.
[8]	Random Forest with accuracy of 96.26%	Total URLs of 18982 from ISCX-URL-2016 dataset.	Classification of 5 classes. The proposed dmodel outperformed DNN model.	Tested on small dataset
[9]	Linear and nonlinear space transformation methods with accuracy of 82%	Total URLs of 331622	Developed a website to demonstrate the detection system	Classification for 2 classes only
[10]	LightGBM with accuracy of 86%.	Total URLs of 38300 from Phish Tank dataset.	The proposed model outperformed RF, DT, regression and SVM methods.	Classification for 2 classes only and tested on limited dataset.
[11]	Logistic regression with accuracy of 91%.	Total URLs of more than 32000 from PhishTank, Kaggle, Github.com	The proposed method outperformed SVM, KNN and LDA methods.	Classification for 2 classes only and tested on limited data.
[12]	Voting ensemble classifier with accuracy of 94.99%	Total URLs of 11056 from Kaggle repository	Applied ensemble classifier methods that outperformed individual classifiers.	Classification for 2 classes only and tested on limited data.
	Stacking ensemble classifier with accuracy of 94.81%			
[13]	Voting Ensemble Classifier with accuracy of 98.27%	Total URLs: 75000	Applied ensemble classifier methods that outperformed individual classifiers.	Classification for 2 classes only
	Stacking Ensemble Classifier with accuracy of 98.23%			

Based on literature review, there has been different work where various machine-learning algorithms applied to classify malicious URLs for network intrusion detection systems. However, previous works mostly were trained for binary classifications of URLs and tested on limited URL datasets. Besides that, previous works were tested on limited URL datasets and encountered challenges in detecting malicious URLs due to the large data volume, unpredictable trends, and complex links between features. Therefore, there is a need to develop a more effective classification model that can perform multi-class classification to detect malicious URLs.

To overcome the above-mentioned challenges, this work proposed a feature engineering approach and stacking-based ensemble framework to perform multi-class classification of malicious URLs on larger URL datasets. The contribution of this work is the incorporation of four machine learning methods namely XGBoost, CatBoost, LightGBM, and Random Forest in a stacking-based framework to classify four classes of malicious URLs. Besides, extensive analysis was performed to justify the significant contribution of the proposed framework compared to previous works.

## 3. Implementation methodology

### 3.1 Feature engineering

The process of feature engineering entails choosing and developing fresh features from the raw data that can be applied to model training. These features can be formed by merging or altering the already present features in the data, or they can be extracted directly from the raw data. To train a model to handle a specific problem, a set of features that are pertinent, instructive, and beneficial must be created.

In general, malicious sites can be classified by obtaining web and URL features that fall into 4 main categories which are domain-based features, host-based features, reputation-based features, and lexical features. This study focuses on obtaining lexical features directly from the URL to identify malicious websites. The textual characteristics of a URL known as lexical features include the hostname and URL lengths, tokens contained in the URL, and other textual information. Lexical features have become one of the most used sources of features in machine learning because of their low computational complexity, safety, and excellent classification accuracy as this approach has been used in other similar studies. Features gathered from URLs are independent of any application, including email, social networking sites, and gaming. Lexical features are still accessible even after a malicious webpage is offline because many fraudulent URLs have a short lifespan [11–13]. An important aspect of feature engineering in this study is to convert the lexical features into numeric attributes since supervised machine learning models can only work using numerical data. In this study, a total of 22 lexical features have been extracted. The features used and their description are provided in Table 2 [14].

**Table 2:** The 22 lexical features were extracted using the feature engineering approach.

Lexical Features	Description
use_of_ip	This feature is used to determine the possibility of the URL having an IP address.
abnormal_url	This feature is by examining the existence of the URL in the WHOIS database.
suspicious_words	Suspicious words such as PayPal, log in, sign in, banks, accounts, updates, bonuses, services, etc. are commonly observed in malicious URLs. This feature signifies whether such suspicious words are commonly repeated in the URL.
digit_count	This feature calculates the number of digits present in a URL. Legit URLs usually do not include many digits. For example, in phishing URLs, masking is achieved by adding digits among the letters.
count_?	This feature is a query string that includes information to be passed to the server. It is denoted by the occurrence of a (?) symbol in the URL.
count@	This feature includes information on @ symbol. The @ symbol can be manipulated by hackers to create malicious URLs.
no_of_dir	This feature calculates the number of directories that exist in the URL. Distrustful websites may have several directories in their URL path.
count-dot	This feature indicates the number of subdomains present where each domain is split by a period/dot.
count-www	This feature counts the number of www in the URL.
google_index	This feature examines whether the URL is indexed in Google search.
count_embedded_domain	This feature calculates the number of (‘//’) that exist in the URL where the quantity of ‘//’ denotes the number of embedded domains
short_url	This feature identifies whether the URL adopts URL shortening services such as bit. \ly, google, etc.
count_https	This is an important feature because malicious URLs do not use https protocol. After all, it expects the user’s authorization.
count_http	Malicious URLs usually practice more than one http protocol.
count%	Legit URLs often have fewer (%) symbols compared to malicious URLs.
count_dash	This feature calculates the number of dashs (-) in the URL.
count_equal	This feature calculates the quantity of equal symbols that exist in the URL.
url_length	This feature calculates the URL length. Hackers often use long URLs to conceal the domain name
hostname_length	This feature denotes the length of the hostname.
first_diretory_length	This feature counts the length of the first directory in the URL.
top_level_domain	This feature calculates the length of top level domain in a URL.
count_letters	This feature counts the number of letters present in a URL.

### 3.2 Data pre-processing

This work involves two pre-processing stages namely label encoding and scaling before the data is fed to the classifier.

#### 3.2.1 Label encoding

The label for this dataset is the type of malicious URL such as benign, malware, phishing, and defacement. Label encoding aims to convert the evidence into a numeric form for the machine learning to perform classification process. Thus, with the use of label encoding, categorical or text data can be transformed into numerical data that can be incorporated into machine learning algorithms. Each category in the data is given a distinct number value as part of the label encoding procedure. For instance, label encoding would change the three categories of a categorical feature in a dataset, "red," "green," and "blue," into numerical values like 0, 1, and 2. However, label encoding does not take into consideration the relative ordering of the categories, which is a crucial point to keep in mind. Label encoding will assign the numbers 0, 1, and 2, for instance, if the categories are "low," "medium," and "high," but these values do not imply any type of ordering [14].

**Fig 1:**
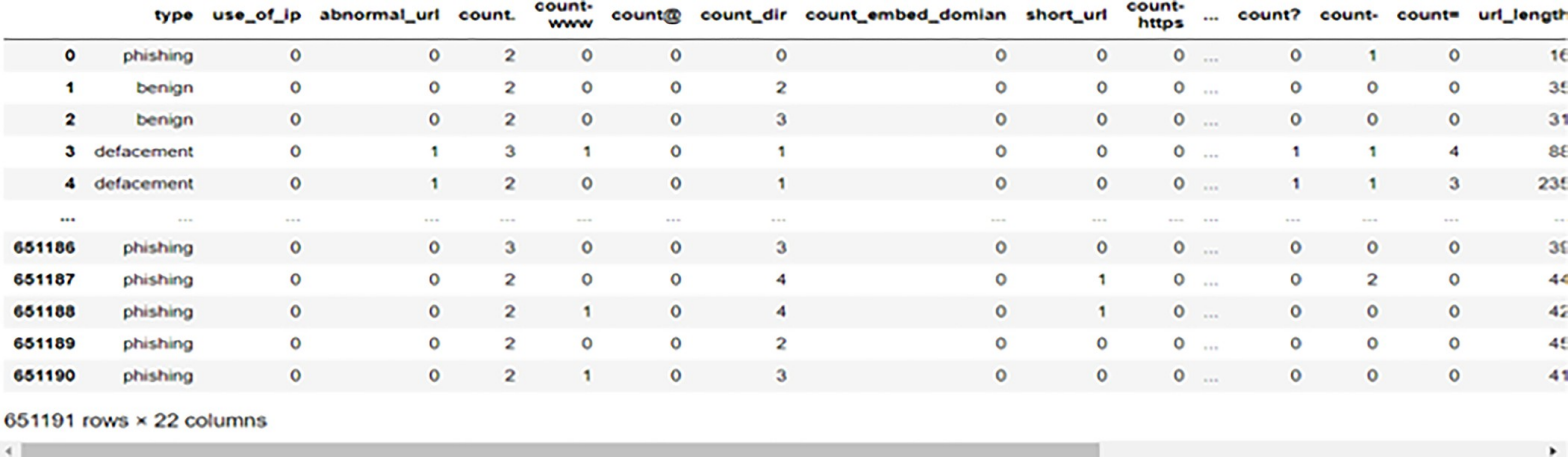
The lexical features of the URLs dataset.

In this study, the categorical data are encoded as follows: benign = 0, defacement = 1, phishing = 2, and malware = 3. Fig 1 presents examples of lexical features extracted from the URLs dataset used in this work. The first row in Fig 1 represents the lexical features stated in Table 2.

#### 3.2.2 Feature scaling

Feature scaling is a method that normalizes the independent features in data. In machine learning, scaling the features to be on a comparable scale is typically a good practice since it can help the model to perform better. With features that are similar in size, some methods, such as gradient descent, can converge more quickly. Generally, scaling will aid in speeding up the machine learning model training process [14]. In this study, the input features are about similar scale except for digit counts which are in the range of tens. Thus, feature scaling was done using the StandardScaler method to scale the dataset and transform the type of scaling done known as standardization that will not be affected by outliers.

#### 3.2.3 Data training

In this study, the dataset was split using stratified sampling. To make sure that the proportion of particular classes or categories in the sample is representative of the proportion of those groups in the population, stratified sampling is a sampling technique that is used. When working with datasets that are imbalanced and have one class or group that is disproportionately underrepresented compared to the others, this might be quite helpful. This was done due to the dataset having class imbalances as all four classes are not of equal proportion in the dataset. Thus, training and validation set was split with 80% of data being used for training and 20% used for validation. This form of dataset splitting was used in other studies as well [7]. The train_test_split method from scikit-learn library was used.

### 3.3 Ethical approval

Ethical and informed consent for data used. No ethical data in this paper.

## 4 Proposed methodology

In this work, a stacking-based ensemble classifier model is developed as the final classifier. The proposed stacking-based ensemble classifier model is proposed to maximize the detection robustness performance in classifying malicious URLs instead of applying individual machine learning classifier. In a stacking classifier, there are base learners like other ensembles, but the outputs of the base learners are then fed into another model known as a meta-learner which aggregates the input of base learners and makes a final prediction. Thus, in a stacking ensemble, a meta-learner makes the final prediction [12, 13]. Generally, the stacking scheme has of two levels which are level 0 and level 1. In level 0, the base models are trained on the training set, and their outputs are kept as new features in a new dataset. The validation set is then fed to the base models, and their predictions are used as inputs to the meta-model.

In this work, the first step in training a stack is to build a training set for the meta-learner. When training data is fed into each base model, for every training instance the base model will provide a prediction in the case of classification task and the base model will output a predicted class. The prediction or output from each base model will be used as the training data for the meta-learner. Once the meta-model has been trained, predictions can be made on novel data where the new data is fed into base learners which will output predictions that will be used as the input data by the meta-learner to make the final prediction. Therefore, by learning how to appropriately combine their predictions, the stacking ensemble classifier integrates the predictions of many base models. As the stacking ensemble classifier can take advantage of the complementing characteristics of various models, it frequently produces higher prediction performance than individual base models. Stacking is especially helpful when the underlying models have different traits or biases since they can make up for one another’s shortcomings. It is also important to note that stacking adds complexity and processing cost because several models must be developed and predictions must be made throughout the inference and training phases [12, 13].

**Fig 2:**
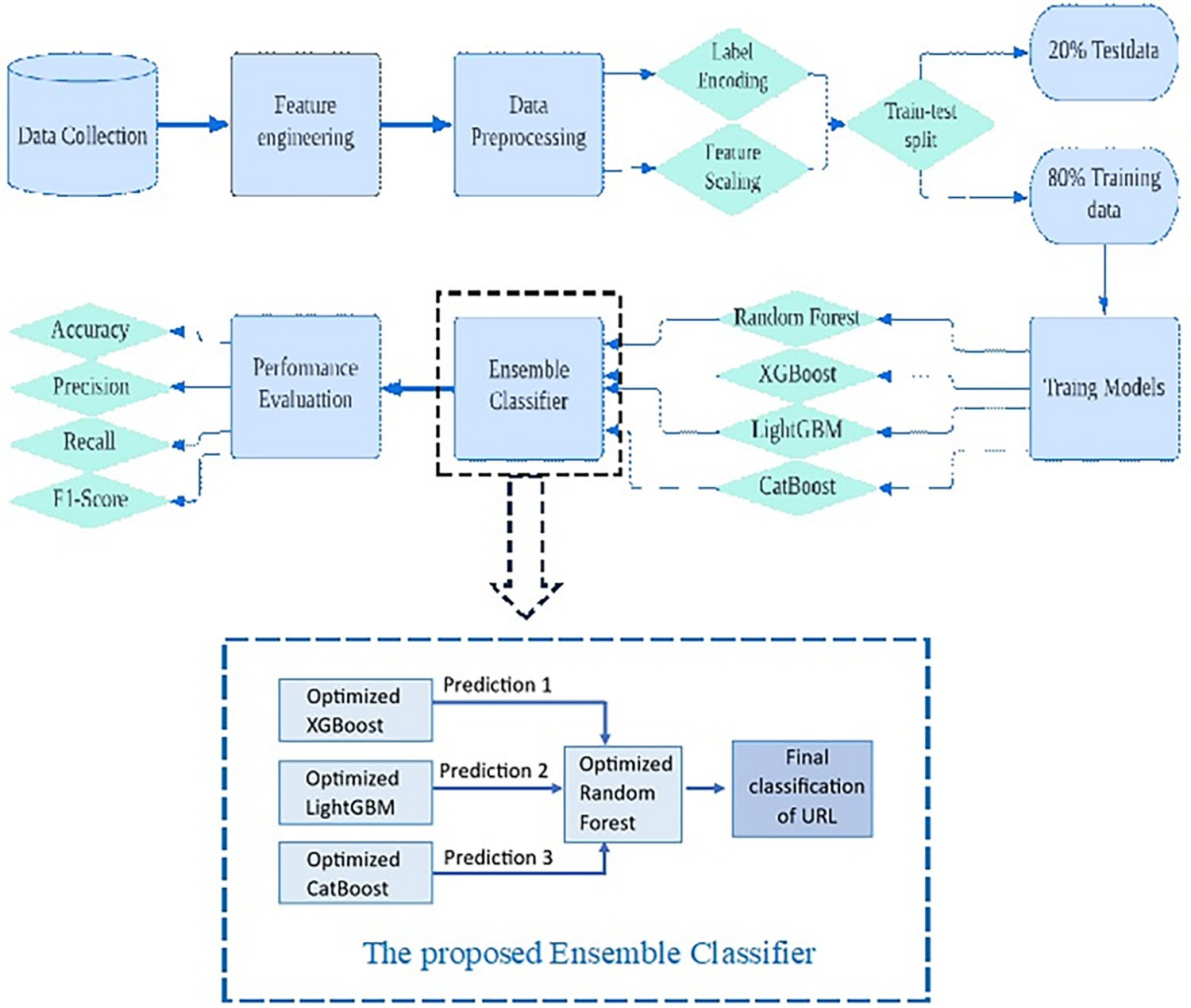
The proposed methodology of this work.

In this study, the boosting algorithms which are XGBoost, CatBoost, and LightGBM are used as the base-learners, and the Random Forest model is trained to be the meta-learner in forming a stacking ensemble classifier. The main motivation behind the training of a stacking classifier is to push the limits of model training to obtain better model accuracy. The proposed methodology of this work with the proposed ensemble classifier as the final classification is depicted in Fig 2.

The algorithm of the proposed work is as below:

Input: Malware Binaries (MB)

Output: Predictions

1. MB ← Dataset (Binaries)
2. For all i ∈ MB do
3. features = feature_extraction(i)
4. End For
5. For all j ∈ features do
6. Base_prediction_1 = Catboost(j)
7. Base_prediction_2 = LightGBM(i)
8. Base_Prediction_3 = XGBoost(i)
9. End for
10. Predicted_outcomes = combine(Base_prediction_1, Base_prediction_2, Base_prediction_3)
11. For all P ∈ Predicted_outcomes do
12. Ensembled_prediction = Random_forest(P)
13. End For
14. Return Ensembled_prediction

### 4.1 Optimized Random Forest

The Random Forest (RF) method aggregates the findings of numerous decision trees by a majority vote. The bootstrap method is applied to select various samples from the initial dataset to generate each decision tree in the forest. The committee then votes on the choices selected by the several individual trees. Finally, the class with the highest votes will be the estimated class. The Classification and Regression Trees (CART) methods and the boost bagging combination method are adopted to generate trees in the RF approach [6].

A dataset, D includes *m* samples, the data is randomly sampled from *m* times to create a new data D’ that has *m* samples. The likelihood of choosing a sample *x* is *1/m* for each sampling round. Samples are chosen from the training data set using the Bootstrap method, which produces data that will build trees and data that will not. Out of all the variables, *m* variables are picked for each node, and the Gini index is computed to produce the best separations. The mathematical calculation of the Gini index is shown in the formula below.


$$Gini(p)=\sum_{k-1}^{k}P_{k}(1-P_{k})$$ (1)

*P_k_* and *k* are the number of classes and occurrences of class *k* in the node, respectively. In the model created using the data that doesn’t grow trees, estimations are made, and estimation errors are computed. The method uses a lot of decision trees to increase classification performance and correct classification rate because each decision tree cannot perform as well when it encounters a different data group than the data group it was trained in. Besides that, the RF model does have an advantage that is feature importance. In addition during training, RF can retrieve the features that the model assigned more weightage in making predictions for each instance [15].

For this study, the Random Forest classifier was used to train model. To optimize the model, hyperparameter tuning was done as shown in Table 3.

**Table 3:** Hyperparameter of tuned Random Forest classifier.

Hyperparameter	Description	Tuned value range(value tuned)
n_estimators	number of decision trees to be trained	50 to 100 (100)
max_depth	to set the depth to which each tree is allowed to grow.	1 to 10 (9)
min_sample_leaf	minimum number of samples required to form a leaf node	1 to 6 (3)
min_sample_split	minimum number of samples needed to split a node	1 to 6 (5)

### 4.2 Optimized Extreme Gradient Boosting (XGBoost)

Extreme Gradient Boosting (XGBoost) utilizes the boosting strategy. Boosting is an iterative procedure in which each new model seeks to improve upon the errors of the prior models. Decision trees are the main base learners in XGBoost. In XGBoost, decision trees are built by beginning from the root and recursively partitioning the data according to specific criteria, like lowering the variance or minimizing the loss function. The decision tree is trained to minimize this gradient by XGBoost, which calculates the gradient of the loss function concerning the predicted values in each iteration [16]. The integrated model can be represented as follows, assuming the model has k decision trees.


$${\hat{y}}_{i}=\sum_{1}^{k}f_{k}(x_{i}),f_{k}\in F$$ (2)

Given: *F* is the set of regression trees, while *f* is a regression tree in the set.

The underlying assumption of the method is that each update is initiated on the previous model’s predicted outcomes. A new model is produced by including a new tree *f* to compute the residual error between the true value and the prior tree’s prediction outcomes, and the new model is then utilized as the foundation for the subsequent model learning. The maximum generalization capability is necessary because the objective of prediction is obviously to bring the predicted value of the number group as close as possible to the true value yi. The objective function is simplified as given below because this is a functional optimization problem from a mathematical perspective.


$${obj}^{\left(t\right)}=l({y_{i},\hat{y}}_{i}^{\left(t\right)})+\Omega (f_{t})$$ (3)

Given: The term $l({y_{i},\hat{y}}_{i}^{\left(t\right)})$ is the loss function and the second term Ω(*f_t_*) is the regularisation term added. Minimization of the loss function will allow optimization of the model to fit the dataset better with less error in prediction and the regularisation parameter adds a penalty to prevent over-optimization of the model that may result in overfitting [15, 16].

The following hyperparameters were tuned by using the Randomized Search method to optimize the XGBoost classifier (Table 4).

**Table 4:** Hyperparameter of XGBoost classifier.

Hyperparameter	Description	Tuned value range (value tuned)
n_estimators	the number of decision trees to be trained	25 to 100 (54)
max_depth	to set the depth to which each tree is allowed to grow	6 to 10 (8)
learning_rate	to alter the model in response to the estimated error	0.05 to 0.2 (0.2)

### 4.3 Optimized Category Boosting (CatBoost)

CatBoost method lessens the requirement for feature preprocessing and aids in the extraction of useful information from categorical data. CatBoost proposes an improvement known as ordered boosting, which makes use of the inherent order of categorical features during training. It considers the order in which categories inside a feature are sorted and preserves that order when building the trees. This method aids the model in capturing significant interactions between categories and more informed category splits [16]. The following hyperparameter was tuned for the classifier to obtain an optimized model as shown in (Table 5).

**Table 5:** Hyperparameter of Catboost classifier.

Hyperparameter	Description	Tuned value range (value tuned)
depth	sets the depth to which each decision tree is allowed to grow	2 to 10 (8)
iterations	defines the number of times the algorithm runs to minimize the loss function.	5 to 20 (14)

### 4.4 Optimized Light Gradient Boosting Machine (LightGBM)

LightGBM is categorized as a gradient-boosting framework that offers effective and high-performance training for huge datasets. For a given dataset, the LightGBM model and its objective function is given below

$${\hat{y}}_{i}=\sum_{1}^{k}f_{k}(x_{i})$$ (4)

Given: *f* is the decision tree and ${\hat{y}}_{i}$ is the predicted value.

The loss function is optimized by LightGBM utilizing a gradient-based method. The prediction provided by the group of decision tree calculates the gradients of the loss function. By updating the prediction in the direction of the negative gradient, the model seeks to minimize the loss. The regularisation term is added to penalize the minimization of loss function from overfitting the model.

LightGBM adopts a tree generation method called leaf-wise tree growth. The leaf node with the highest split gain among the leaf nodes is chosen as the subsequent split leaf node which results in maximum reduction of loss function. The leaf-wise method is more accurate and can reduce errors when dividing the tree for the same number of times as the level-wise method. As a result, the decision tree might become overfit. To avoid overfitting and maintain high efficiency, LightGBM includes a maximum depth limit. To make the data expression simpler and use less memory, LightGBM uses histogram optimization to split continuous values into several discrete domains. Additionally, histogram regularisation helps the model to avoid overfitting and produce better generalization outcomes. LightGBM employs two algorithms to minimize the size of the feature set and dataset which are exclusive feature bundling (EFB) and gradient-based one-sided sampling (GOSS). To calculate the information gain while being as consistent with the total data distribution as possible and making sure that samples with small gradient values are trained, GOSS uses samples with high and small gradients. To cut down on feature dimensions and boost computing performance, EFB groups together features that are mutually exclusive [3].

The following hyperparameter was tuned to optimize the Light GBM classifier (Table 6).

**Table 6:** Hyperparameter of LightGBM classifier.

Hyperparameter	Description	Tuned value range (value tuned)
n_estimators	the number of decision trees to be trained	20 to 100 (94)
max_depth	to set the depth to which each tree is allowed to grow	6 to 10 (8)
learning_rate	to alter the model in response to the estimated error	0.05 to 0.2 (0.2)

## 5 Results and discussions

In this work, a stacking-based ensemble classifier is developed to classify the URLs into four classes namely benign, defacement, phishing, and malware. The main purpose of training an ensemble model is to exploit the performance of individual classifiers by combining them to build a stronger classifier. The dataset was split into 80% training and 20% testing. This work is performed using a Windows 11 PC with AMD Ryzen 7 5700U CPU @ 1.8GHz and 8GB DDR4 @ 3200MHZ of memory.

Initially, the performance of the proposed ensemble classifier model was evaluated on the training dataset. Generally, a high training score indicates that the model has a low bias. Then, five-fold cross-validation was performed to observe the generalization capability of the model and the average validation score obtained was 98%. The high validation score indicates that the model is not overfitting the dataset and will be able to generalize well.

In a classification task, precision is a metric for a classifier’s accuracy. It is known as the proportion of positive cases that are anticipated to be positive. Precision in this study will indicate how well the classifier performs at classifying each instance in its respective class correctly. For instance, if 100 URLs were classified as malware then precision would be the percentage of URLs out of 100 that is malware.

$$Precision=\frac{True\;Positive}{True\;Positive+False\;Positive}$$ (5)

where True Positive (TP) denotes the number of successfully detected malicious URL by the detection model and False Positive (FP) denotes the number of falsely detected malicious URL by the detection model.

Meanwhile, recall serves as a gauge of how comprehensive a classifier is. It is measured as the percentage of cases that were classified as positive and that the classifier properly anticipated. For instance, in this study, there are 32520 malware URLs then recall is the measure of how many of those URLs will be correctly classified. In other words, it reflects on the ability of the classifier to capture most of the true positive cases.

$$Recall=\frac{True\;Positive}{True\;Positive+False\;Negative}$$ (6)

where False Negative (FN) refers to the number of undetected malicious URL.

In theory, a good classifier will be one with high precision and high recall. However, increasing the precision will decrease the recall and vice versa. This scenario is known as a precision-recall trade-off. A metric that combines recall and precision is the F1 score [17]. Based on the results in Table 7, the proposed ensemble classifier performs well in classifying the four classes of malicious URLs with precision ranging between 91% to 99% while recall value ranging between 87% to 99%. The average accuracy of the proposed method in classifying four classes of malicious URLs is 96.8%. As can be seen, malware has the lowest recall and F1-score of 87% and 89% respectively because malware is the most challenging to detect compared to other types of malicious URLs. It is difficult to detect malware because malware usually appears as normal URLs and go unnoticed by users hence enable it to steal users’ information.

**Table 7:** The performance of the proposed ensemble classifier in classifying four classes of malicious URLs for testing data.

Class	Precision (%)	Recall (%)	F1-score (%)	Accuracy (%)
Benign	97	99	99	
Defacement	99	99	99	96.8
Phishing	96	94	95	
Malware	91	87	89	

**Fig 3:**
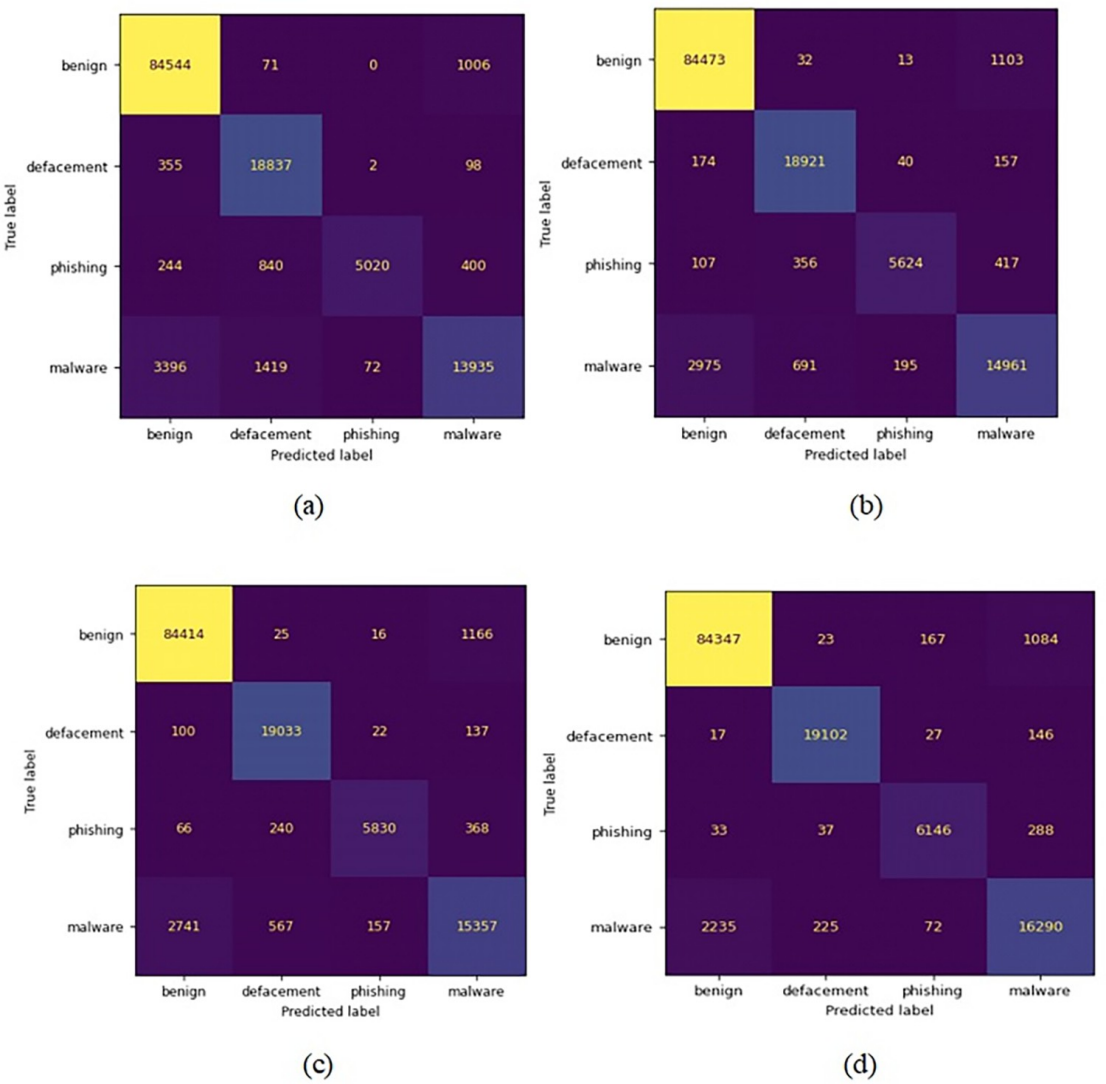
Confusion matrix of the (a) Random Forest, (b) XGBoost, (c) LightGBM, and (d) proposed ensemble classifier.

Fig 3 presents four confusion matrices to explain the effectiveness of the classification models in classifying the malicious URLs. A confusion matrix provides an overview of the model’s predictions, contrasting the predicted values with the actual values. It enables us to see the model’s performance and errors occurring. Most of the benign URLs are misclassified as malware followed by phishing and defacement. As can be seen in the confusion matrix of the proposed ensemble classifier, 1.3% (1084 URLs) of the benign URLs are misclassified as malware URLs. For malware URL test data, approximately 12% (2235 URLs) of malware URLs are misclassified as benign which shows that malware has a considerably high possibility of being misclassified as benign due to its complex features. This shows that malware URLs and benign URLs have almost similar features which requires an effective feature extraction method and accurate classifier to differentiate both classes.

**Fig 4:**
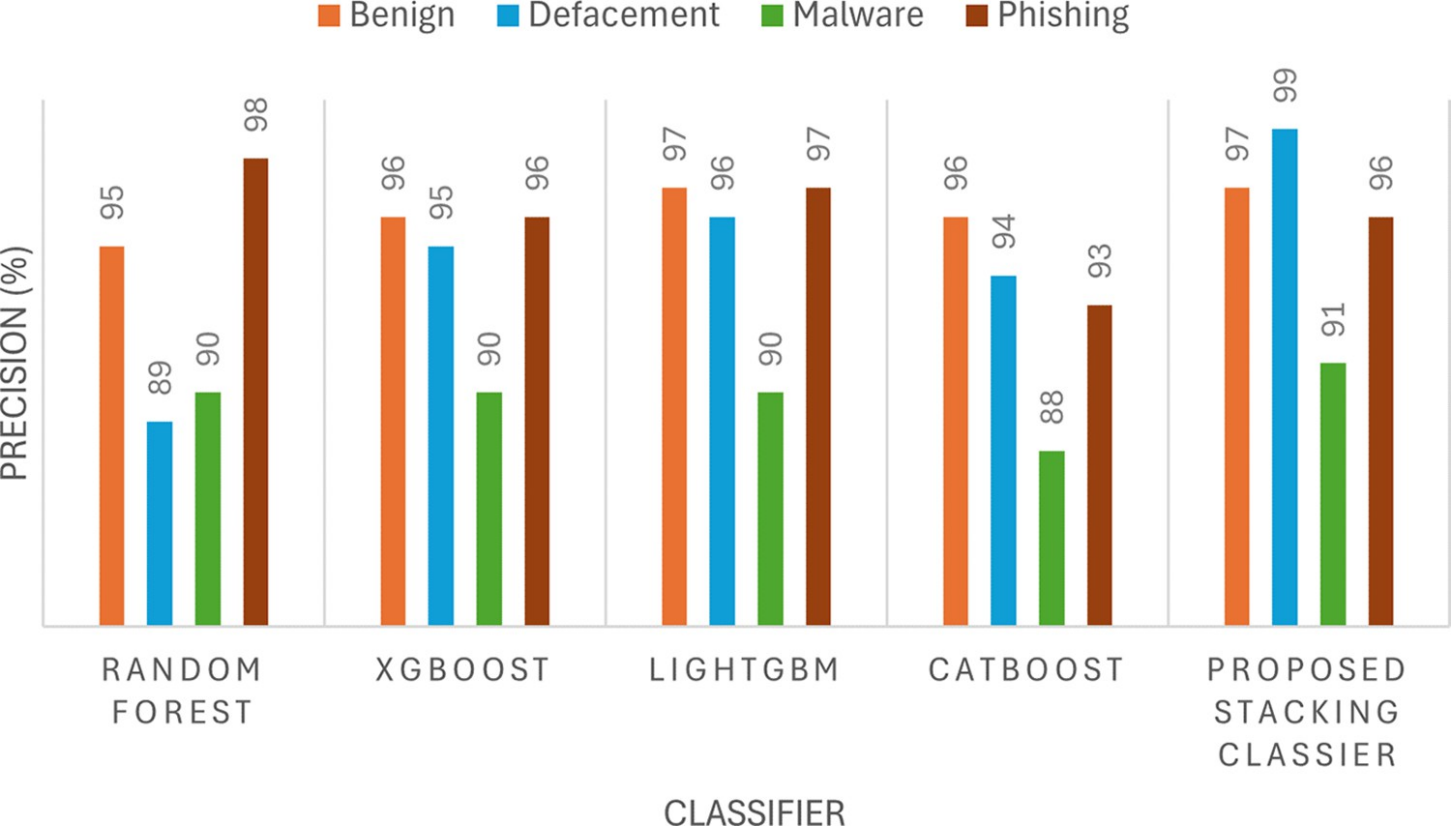
Performance of several classification methods in terms of precision value.

**Fig 5:**
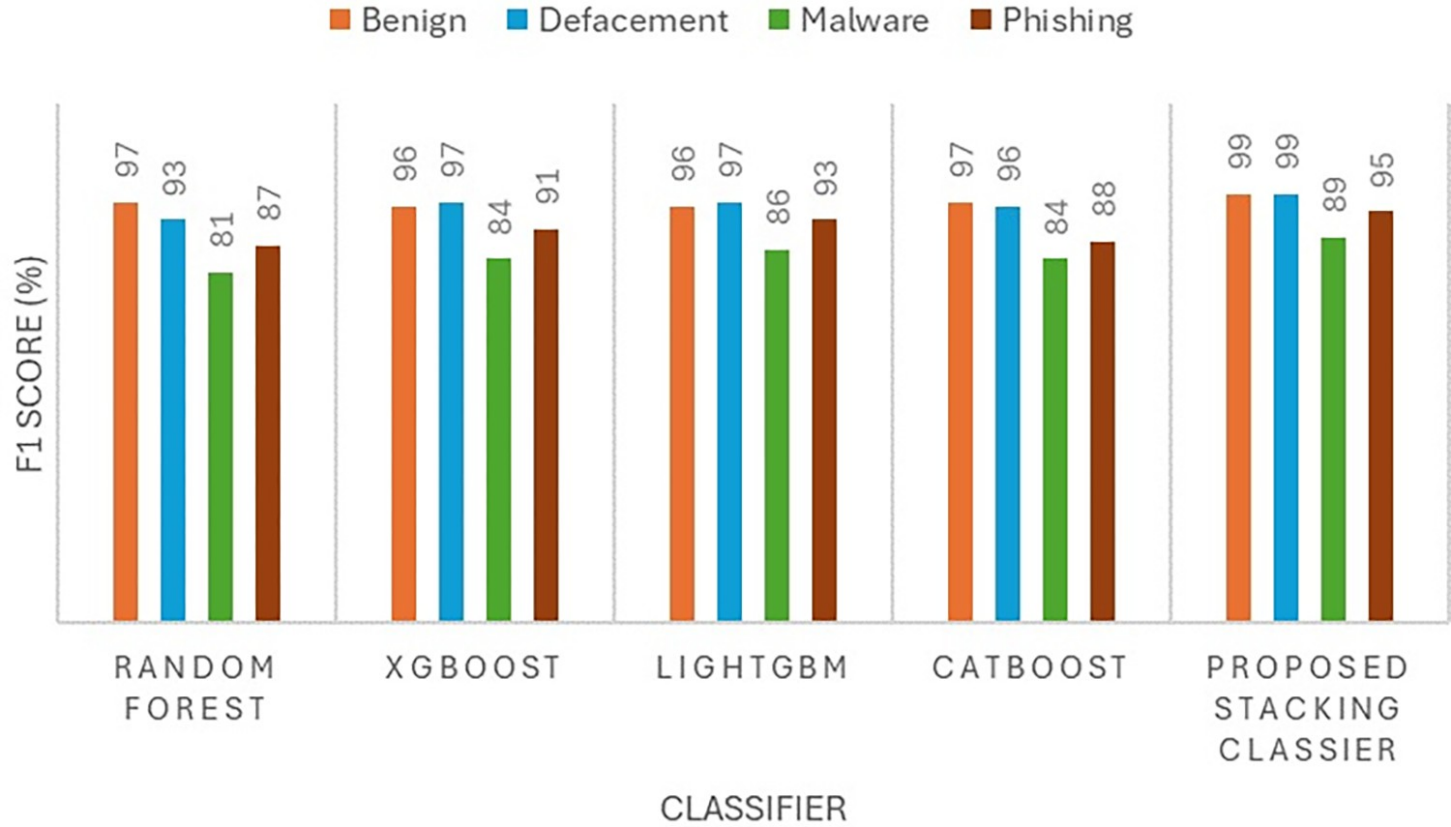


**Fig 6:**
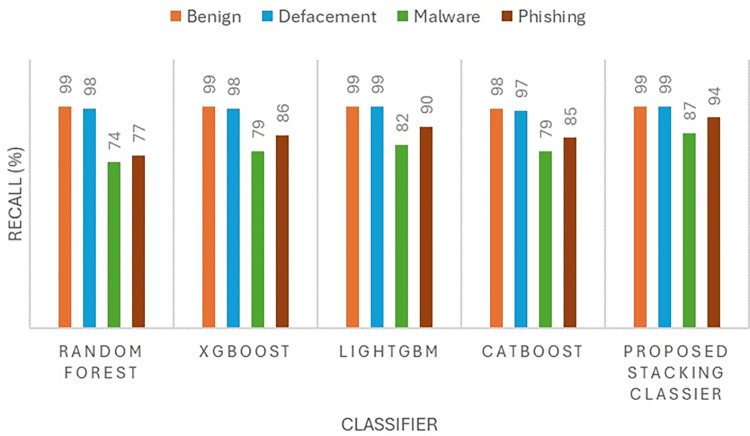


**Fig 7:**
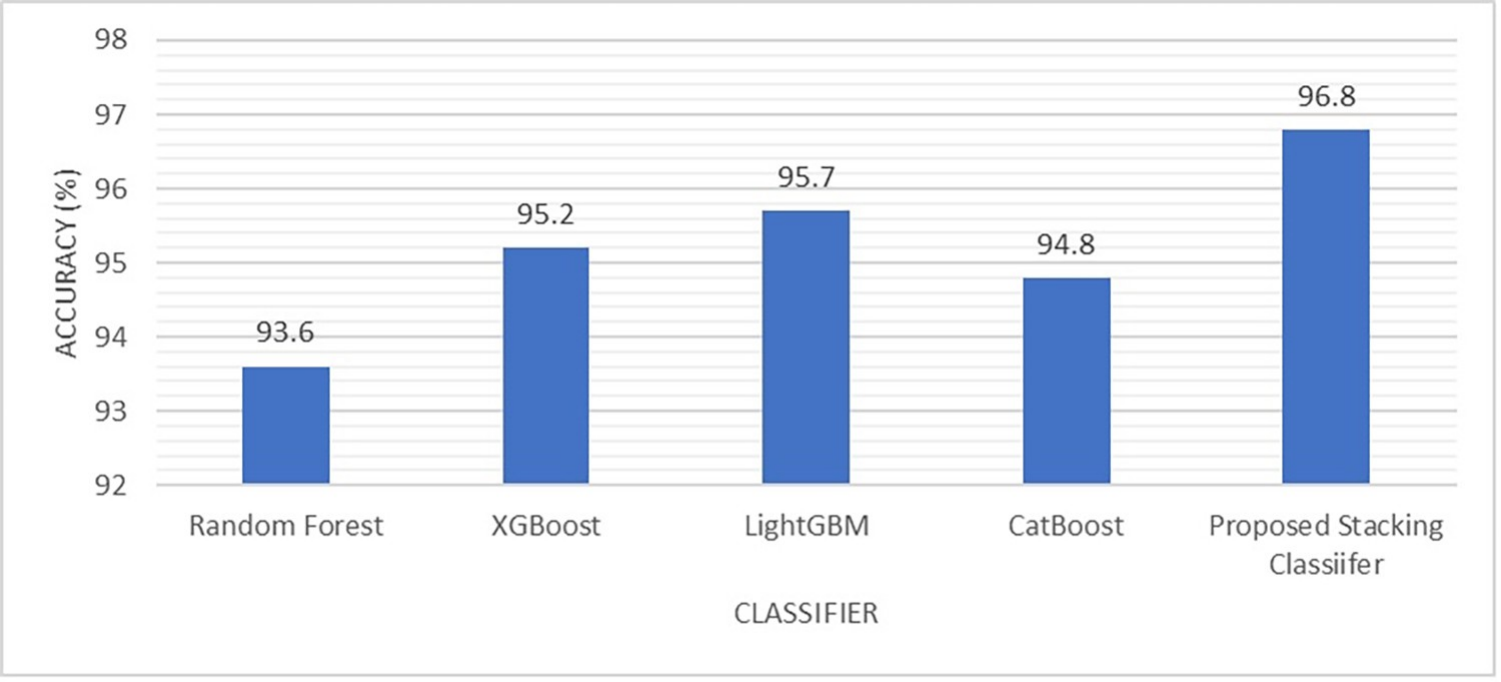
Comparison of the proposed method with several classification methods in terms of average classification accuracy.

The performance of the proposed model is evaluated by using four metrics namely precision, recall, accuracy, and F1-score as presented in Figs 4 to 7. The precision (Fig 4) and F1-score (Fig 5) show the effectiveness of the proposed stacking ensemble classifier compared to other methods namely random forest, XGBoost, LightGBM, and CatBoost. In this study, the four metrics (precision, recall, accuracy, F1-score) are used to assess the performance of the classifier.

In terms of phishing URLs class, the proposed ensemble stacking classifier achieved a recall of 94% (Fig 6). The recall achieved by the proposed ensemble classifiers is higher than the best individual classifier which is LightGBM that achieved a recall of 90% for phishing URLs. Besides that, the recall for phishing by ensemble learners is much higher than other individual classifiers such as Random Forest, XGBoost and CatBoost which had a recall of 77%, 86% and 85% respectively. Apart from that, the malware class is examined where the proposed stacking classifier achieved a recall of 87%. The recall achieved by the proposed stacking ensemble is the highest for the malware class compared to individual classifiers. The second highest recall is achieved by LightGBM model with 82% recall. The 5% improvement in malware classification is significant whereby referring to Fig 3, it can be calculated that the proposed stacking classifier was able to classify 933 malware URLs more than LightGBM classifier correctly. Furthermore, comparing the recall to the second best individual classifier, XGBoost which achieved a recall of 79 percent for malware URLs, there is 8% improvement when applying the proposed stacking classifier. Besides that, comparing the Random Forest and CatBoost methods that achieved a recall of 74% and 79% respectively, the proposed stacking ensemble showed an improvement in recall by 13% and 8% respectively. Thus, the recall achieved by the proposed stacking classifier is the highest in this study for malware URLs which is at 87%. Thus, from the analysis it can be understood that the proposed ensemble model has better classification of phishing and malware URLs than each individual base model in this study. However, it should be noted the proposed ensemble classifier still classified around 12% of malware URLs as benign which is less than the misclassification by LightGBM model. LightGBM model has malware misclassification of 15% as benign. Therefore, it can be deduced that in terms of recall rate the proposed ensemble classifiers has achieved better performance than all four individual classifiers used in this study which satisfies the aim of training the ensemble model.

Based on Figs 4 and 6, it can be observed that XGBoost model achieved high precision and recall for the class of benign and defacement URLs with both values being over 95%. By taking only the recall for benign and defacement into account, their values are 99% and 98% respectively, which clearly indicates that XGBoost classifier is performing very well at capturing most of the benign and defacement URLs from the test dataset. It should be noted that, comparing the performance of XGBoost to Random Forest in terms of benign and defacement URLs classification, the performance is almost the same with minor difference in precision. As for Random Forest classifier, the performance on phishing URLs was deduced that the classifier had a rather poor recall at 77% where most of the misclassified phishing URLs at about 13% were misclassified as defacement URLs. On the other hand, the XGBoost classifier has shown significant improvement over Random Forest where the recall for phishing URLs has been 86% and the misclassification of phishing as defacement has fallen from 13% to 5%. This shows that XGBoost classifier was able to distinguish better between phishing and defacement URLs compared to Random Forest. However, both classifiers still misclassified 6% of phishing URLs as malware thus, the XGBoost classifier has the same limitation of the Random Forest when it comes to distinguishing between phishing and malware URLs. Despite that, the XGBoost classifier certainly performs better in terms of classifying phishing URLs compared to Random Forest.

Recall and accuracy are most significant metrics because the recall indicates the number of malicious URLs that were captured from the overall dataset since having a false negative scenario is more risky than false positive. This is because malicious URLs that fail to be captured will pose a bigger threat than benign URLs that were wrongly classified as malicious [18–20]. Besides that, accuracy also indicates the reliability of the classifier in detecting novel malicious URLs. Fig 6 shows the significant improvement in terms of recall value when applying the proposed stacking classifier in classifying the four types of malicious URLs compared to other methods namely random forest, XGBoost, LightGBM, and CatBoost. As can be seen, Malware has the lowest recall for all five classifier methods which means that malware is the most challenging to detect compared to other types of malicious URLs. Fig 7 presents the accuracy of individual machine learning methods and the proposed stacking method. The proposed stacking method which incorporates the four machine learning models (XGBoost, LightGBM, CatBoost, and random forest) achieves the highest accuracy compared to applying the machine learning model individually.

The improvement in the performance justifies the contribution of the extracted features obtained from the feature engineering method and the ensemble classifier. The proposed ensemble classifier overcomes the limitations encountered by individual classifiers which resulted in accurate classification of malicious URLs. Besides that, the feature engineering method managed to extract discriminant features of the URLs which were then fed to the proposed classifier.

One advantage of the proposed ensemble classifier is that it improved the predictive performance of the malicious URLs detection compared to individual learners by using multiple learning algorithms. Another advantage of the proposed ensemble classifier is that ensemble training method can improve the robustness of machine learning systems against adversarial attacks. The applied ensemble method is very useful when the URL dataset consist of both linear and non-linear type of data. The ensemble classifier can also reduce the risk of overfitting and underfitting by balancing the trade-off between bias and variance. However, the proposed ensemble learning method can have poor scalability when containing more sub-models in the ensemble. Besides that, it can be computationally expensive and time-consuming to execute ensemble classifier due to the need for training and storing multiple machine learning models, and integrating their outputs.

Table 8 benchmarks the performance of the proposed method with previous works in classifying malicious URLs using different URLs dataset. Despite the different dataset adopted in previous works, all the works investigated in Table 8 focused malicious URLs classification. The comparison is tabulated in Table 8 review the methods that have been applied in previous works and the accuracy associated the methods applied. As can be seen, most of the previous works [3, 4, 6, 7, 9–13], focused on the binary classification of malicious URL detection. Detecting multi-class malicious URLs is more challenging compared to binary classification since it requires rigorous feature extraction and classification to identify the types of malicious URLs. Albeit the high accuracy obtained in [4, 5, 13], the works in [4, 13] focused on binary classification only instead of multi-class classification while the work in [5] includes limited data of 57,000 URLs only. Most of previous works applied much lesser data compared to this study with 651191 URLs which offers better generalisation. The work in [12] also proposed a stacking classifier method, however, the work in [12] focused on binary classification with a limited number of URLs. Meanwhile, the work in [20] adopted random forest method to classify malicious URLs with accuracy of 96.6%. In contract, when this work applied random forest method, the classification accuracy achieved is 93.6%. On the other hand, this work focused on multi-class classification and included larger data with a total of 651,191 URLs. This work also evaluated the performance of several machine learning models namely random forest, XGBoost, LightGBM, and CatBoost an the classification accuracies obtained are 93.6%, 95.2%, 95.7% and 94.8% respectively. The proposed stacking-based ensemble classifier has shown significant results in classifying four classes of malicious URLs (phishing, malware, defacement, and benign) with an average accuracy of 96.8% when benchmarked with previous works. Based on Table 8, the performance of the proposed model shows better performance compared to other state-of-the-art models.

**Table 8:** Benchmarking the proposed work with previous works in malicious URL detection.

Work	Method	Classification	Accuracy
[3]	LightGBM	Phishing and non-phishing	93.88%
[4]	Deep Learning	Malicious and Genuine	99.60%
[5]	Bagging Decision Tree Ensemble	Benign, Phishing, Malware, Spam, Defacement	97.92%
[6]	Logistic Regression SVM Random Forest	Phishing and legitimate	90.28% 93.43% 95.51%.
[7]	KNN using random subspace technique	Malicious and benign	89.24%
[8]	Random Forest	Benign, Phishing, Malware, Spam, Defacement	96.26%
[9]	Linear and nonlinear space transformation methods	Benign, Malicious	82%
[10]	LightGBM	Phishing and benign	86.0%
[11]	Logistic regression	Benign, Malicious	91%
[12]	Voting ensemble classifier	Benign and Phishing	94.99%
	Stacking ensemble classifier		94.81%
[13]	Voting Ensemble Classifier	Benign and Phishing	98.27%
	Stacking Ensemble Classifier		98.23%
[20]	Random forest	Benign, Phishing, Malware, Defacement	96.6%
This work	Random Forest, XGBoost LightGBM CatBoost	Benign, Phishing, Malware, Defacement	93.6%, 95.2%, 95.7% 94.8%
**This work**	**Proposed stacking ensemble classifier**	**Benign, Phishing, Malware, Defacement**	**96.8%**

## 6 Conclusion & future work

In this study, a total of 651191 URLs have been used as the dataset where a total of 22 lexical features have been extracted via the feature engineering method. This work proposed a stacking-based ensemble framework by incorporating four machine learning models namely Random Forest, XGBoost, LightGBM, and CatBoost to classify malicious URLs. The hyperparameters of the proposed ensemble classifier are tuned using the Randomized Search method to optimize the proposed ensemble classifier. The proposed method has shown significant results in classifying four classes of malicious URLs such as benign, phishing, malware, and defacement with an average accuracy of 96.8%. The proposed work has shown significant improvement when benchmarked with previous works.

The future work for this study would focus on improving the recall for each class label especially malware and phishing class as well as improving overall accuracy. In addition, the feature engineering method can be improved by engineering more features from the URLs. More lexical and host-based features can be engineered as this will increase the dimensionality of the data thus providing more valuable insight on detectable patterns in the dataset allowing the machine learning model to fit the data better. Besides that, the number of base classifiers with building ensembles can be increased as more powerful base classifiers can result in better ensemble models. The main motivation behind this study is the importance of cybersecurity in a more connected world, as hacking and online scams are becoming more prevalent. Thus there is a need to identify these malicious sites and protect the internet users.

## Supporting information

S1 File (DOCX)
