# Supplementary material for: An ensemble classification method based on machine learning models for malicious Uniform Resource Locators (URL)
Source: PLoS One. 2024 May 31;19(5):e0302196. doi: 10.1371/journal.pone.0302196 (PMC11142511; doi:10.1371/journal.pone.0302196)
Supplement: S1 File — (DOCX) [file pone.0302196.s001.docx]

from numpy import mean

from numpy import std

import lightgbm as lgb

import xgboost as XGBRFClassifier

import catboost at Catboost

import randomforestclassifier as RF

from sklearn.datasets import make_classification

from sklearn.model_selection import cross_val_score

from sklearn.model_selection import RepeatedStratifiedKFold

# define dataset

df = pd.read_csv('/dataset/malware.csv')

X, y = make_classification(n_samples=1000, n_features=20, n_informative=15, n_redundant=5, random_state=7,df)

def XGBRFClassifier(alg,n_estimators,subsample,colsample_bynode, cv_folds=5, early_stopping_rounds=50,loss_function='CrossEntropy'):

xgb_param = alg.get_xgb_params()

df.xgtrain = xgb.DMatrix(dtrain[predictors].values, label=dtrain[target].values)

cvresult = xgb.cv(xgb_param, xgtrain, num_boost_round=alg.get_params()['n_estimators'], nfold=cv_folds,

metrics='auc', early_stopping_rounds=early_stopping_rounds, show_progress=False)

alg.set_params(n_estimators=cvresult.shape[0])

#Fit the algorithm on the data

alg.fit(dtrain[predictors], dtrain['Disbursed'],eval_metric='auc')

#Predict training set:

dtrain_predictions = alg.predict(dtrain[predictors])

dtrain_predprob = alg.predict_proba(dtrain[predictors])[:,1]

feat_imp = pd.Series(alg.booster().get_fscore()).sort_values(ascending=False)

feat_imp.plot(kind='bar', title='Feature Importances')

plt.ylabel('Feature Importance Score')

return feat_imp

def LGBMClassifier(boosting_type='gbdt', class_weight=None, colsample_bytree=1.0,

importance_type='split', learning_rate=0.1, max_depth=-1,

min_child_samples=20, min_child_weight=0.001, min_split_gain=0.0,

alg,loss_function='CrossEntropy'):

lgb_param = alg.get_xgb_params()

df.lgtrain = lgb.DMatrix(dtrain[predictors].values, label=dtrain[target].values)

cvresult = lgb.cv(xgb_param, xgtrain, num_boost_round=alg.get_params()['n_estimators'], nfold=cv_folds,

metrics='auc', early_stopping_rounds=early_stopping_rounds, show_progress=False)

alg.set_params(n_estimators=cvresult.shape[0])

#Fit the algorithm on the data

alg.fit(dtrain[predictors], dtrain['Disbursed'],eval_metric='auc')

#Predict training set:

dtrain_predictions = alg.predict(dtrain[predictors])

dtrain_predprob = alg.predict_proba(dtrain[predictors])[:,1]

feat_imp = pd.Series(alg.booster().get_fscore()).sort_values(ascending=False)

feat_imp.plot(kind='bar', title='Feature Importances')

plt.ylabel('Feature Importance Score')

return feat_imp

def CatBoostClassifier(iterations=5, learning_rate=0.1, loss_function='CrossEntropy',alg,loss_function='CrossEntropy')

cat_param = alg.get_xgb_params()

df.lgtrain =cat.DMatrix(dtrain[predictors].values, label=dtrain[target].values)

cvresult = cat.cv(xgb_param, xgtrain, num_boost_round=alg.get_params()['n_estimators'], nfold=cv_folds,

metrics='auc', early_stopping_rounds=early_stopping_rounds, show_progress=False)

alg.set_params(n_estimators=cvresult.shape[0])

#Fit the algorithm on the data

alg.fit(dtrain[predictors], dtrain['Disbursed'],eval_metric='auc')

#Predict training set:

dtrain_predictions = alg.predict(dtrain[predictors])

dtrain_predprob = alg.predict_proba(dtrain[predictors])[:,1]

feat_imp = pd.Series(alg.booster().get_fscore()).sort_values(ascending=False)

feat_imp.plot(kind='bar', title='Feature Importances')

plt.ylabel('Feature Importance Score')

return feat_imp

# define the model

XGB = XGBRFClassifier(n_estimators=100, subsample=0.9, colsample_bynode=0.2)

LGB = lgb(n_estimators=100, n_jobs=-1, num_leaves=31, objective=None,

random_state=None, reg_alpha=0.0, reg_lambda=0.0, silent=True,

subsample=1.0, subsample_for_bin=200000, subsample_freq=0)

# define the model evaluation procedure

predict = RF.RepeatedStratifiedKFold(n_splits=10, n_repeats=3, random_state=1)

# evaluate the model and collect the scores

n_scores = RF.cross_val_score(XGB, LGB, CAT, X, y, scoring='accuracy', cv=cv, n_jobs=-1)

#Print model report:

print "\nModel Report"

print "Accuracy : %.4g" % metrics.accuracy_score(dtrain['Disbursed'].values, dtrain_predictions)

print "Precision : %.4g" % metrics.precision_score(dtrain['Disbursed'].values, dtrain_predictions)

print "Recall : %.4g" % metrics.recall_score(dtrain['Disbursed'].values, dtrain_predictions)

print "F-score : %.4g" % metrics.F_score(dtrain['Disbursed'].values, dtrain_predictions)

print "AUC Score (Train): %f" % metrics.roc_auc_score(dtrain['Disbursed'], dtrain_predprob)
